# Concerning Diphtheria

**Published:** 1878-04

**Authors:** 


Concerning Diphtheria.—This widely prevalent disease is one of the leading topics in medical journals at present, and we need make no apology for the prominence given to it in our own columns. The modes of treatment suggested are of the most varied character, and time alone can show which are, on the whole, the best. "The survival of the fittest" is pretty certain in the long run. Dr. A. Bachelder, of Pelham, N. H., sends us the following brief note on this subject:

"I wish to suggest to physicians, in treating diphtheria, to use internally a very weak solution of carbolic acid, and for the throat or fauces a solution of hydrochloric acid, about the strength of strong cider vinegar. I have treated every case successfully, so far, with the above-named remedies. Croup is relieved instantly with the acid solution. As far as my experience goes, the last-named remedy stops all morbid development in the throat as surely as the hoe will stop pig-weeds in a hot, sunny day. Apply it to the throat with a brush or sponge, or use as a gargle."

A correspondent in Alabama writes that in a case of diphtheria in his own family, he took equal parts of black pepper and common salt, pulverized them finely, and with a mop applied the mixture directly to the throat. "The effect was magical," and the patient rapidly recovered.

Another correspondent in New York says that he has often tried successfully the method recommended by Dr. Field in an English medical journal, of gargling the throat with a solution of liver of sulphur, or letting the patient inhale the fumes of burning sulphur.—*Boston Journal of Chemistry.*

